# Multiple recombinant dengue type 1 viruses in an isolate from a dengue patient

**DOI:** 10.1099/vir.0.83122-0

**Published:** 2007-12

**Authors:** John Aaskov, Katie Buzacott, Emma Field, Kym Lowry, Alain Berlioz-Arthaud, Edward C. Holmes

**Affiliations:** 1 Institute of Health and Biomedical Innovation, Queensland University of Technology, Brisbane, Australia; 2 Institute Pasteur, Noumea, New Caledonia; 3 Center for Infectious Disease Dynamics, Department of Biology, The Pennsylvania State University, Mueller Laboratory, University Park, PA 16802, USA; 4 Fogarty International Center, National Institutes of Health, Bethesda, MD 20892, USA

## Abstract

Between 2000 and 2004, dengue virus type 1 (DENV-1) genotypes I and II from Asia were introduced into the Pacific region and co-circulated in some localities. Envelope protein gene sequences of DENV-1 from 12 patients infected on the island of New Caledonia were obtained, five of which carried genotype I viruses and six, genotype II viruses. One patient harboured a mixed infection, containing viruses assigned to both genotypes I and II, as well as a number of inter-genotypic recombinants. This is the first report of a population of dengue viruses isolated from a patient containing both parental and recombinant viruses.

Dengue is a major cause of mortality and morbidity in the tropics (Gubler & Meltzer, 1999), and the ability of positive-sense RNA viruses, such as dengue virus (DENV), to evolve rapidly (Craig *et al.*, 2003; Sittisombut *et al.*, 1997; Thu *et al.*, 2004; Wang *et al.*, 2002; Wittke *et al.*, 2002) poses a serious challenge to the development and efficacy of vaccines. The principal source of the genetic diversity in DENV is error-prone replication with RNA-dependent RNA polymerase (Steinhauer *et al.*, 1992), such that one genomic mutation occurs in nearly every cycle of virus replication. DENV populations at a particular locality may also change rapidly due to periodic selective sweeps (Bennett *et al.*, 2003), to the introduction of exotic strains of virus (A-Nuegoonpipat *et al.*, 2004; Messer *et al.*, 2003; Rico Hesse *et al.*, 1997; Thu *et al.*, 2004), to random genetic bottlenecks (Sittisombut *et al.*, 1997; Thu *et al.*, 2004; Wittke *et al.*, 2002) or to intra-serotypic recombination (AbuBakar *et al.*, 2002; Domingo *et al.*, 2006; Holmes *et al.*, 1999; Tolou *et al.*, 2001; Worobey *et al.*, 1999). Whilst it is widely accepted that another positive-strand RNA virus, polio virus, undergoes both intra-serotypic (Ledinko, 1963) and inter-serotypic (Minor *et al.*, 1986; Tolskaya *et al.*, 1983) recombination, as well as inter-species recombination (Kew *et al.*, 2002), there has been considerable debate about whether recombination occurs in DENV (Rico-Hesse, 2003) and the relevance of any recombination to the deployment of live-attenuated flavivirus vaccines (Monath *et al.*, 2005; Seligman & Gould, 2004). However, there is no mechanistic reason why recombination cannot occur in DENV and this process is being described with increasing frequency in hepatitis C virus, another member of the family *Flaviviridae* (Bernardin *et al.*, 2006; Gao *et al.*, 2007; Kageyama *et al.*, 2006; Legrand-Abravanel *et al.*, 2007; Moreno *et al.*, 2006; Noppornpanth *et al.*, 2006).

The ecological conditions most likely to facilitate recombination in DENV involve the co-circulation of multiple viral populations, including distinct genotypes, so that there are opportunities for a mosquito vector to ingest multiple variants by feeding on a number of infected hosts, or for a host to be infected almost simultaneously by vectors infected with different viruses. These opportunities exist throughout most of south-east Asia. Further, as there are numerous reports of multiple serotypes of DENV recovered from single hosts (Gubler *et al.*, 1985; Laille *et al.*, 1991; Twiddy *et al.*, 2002; Wang *et al.*, 2003), it is likely that mixed infections with different genotypes of the same serotype occur where they co-circulate, but that current diagnostic procedures are unable to detect such mixtures. An opportunity for frequent mixed infections with different strains of DENV arose after 2000, when multiple genotypes of DENV-1 were introduced into the Pacific from distant Asian localities, including the Philippines, Myanmar/Thailand and Malaysia (A-Nuegoonpipat *et al.*, 2004).

To determine the origin of the DENV-1 circulating in New Caledonia during an outbreak in 2002 and 2003, virus was first recovered from 12 patients by culturing 100 μl serum on 25 cm^2^ monolayers of C6-36 *Aedes albopictus* cells in 7–8 ml RPMI 1640 tissue-culture medium at 30 °C for 7 days. RNA was recovered from virus in supernate from the infected cultures of C6-36 cells and from the serum of patient D1.New Caledonia.416/03 by using QIAamp viral RNA mini columns (Qiagen) according to the manufacturer's instructions. The envelope (E) protein gene was amplified by RT-PCR (A-Nuegoonpipat *et al.*, 2004) using oligonucleotide primers D1-764F and D1-2467R (see Supplementary Table S1, available in JGV Online), corresponding to regions of the pre-membrane and non-structural 1 proteins of DENV-1, and a mixture of *Taq* and *Pwo* polymerases (Expand Long Template DNA polymerase; Roche). The PCR product was analysed on 1.5 % (w/v) agarose–Tris/acetate EDTA gels and the band of cDNA of interest was excised and purified by using a High Pure PCR Purification kit (Roche) according to the manufacturer's instructions. Approximately 100 ng cDNA or 600 ng plasmid (see below) was sequenced, using 6.4 pmol oligonucleotide primer and an ABI PRISM Dye Terminator cycle sequencing ready reaction kit (Perkin Elmer) according to the manufacturer's instructions. The product was purified by using Dye-EX spin columns (Qiagen) and analysed at the Australian Genome Research Facility on a 3730XL nucleotide sequencer (Applied Biosystems). Sequences are identified as DENV serotype, country of origin, sample number (and clone number)/year sample obtained.

To determine the DENV-1 genotype of each of 12 New Caledonian viruses, we conducted a maximum-likelihood (ML) phylogenetic analysis of the complete E gene (1485 bp) by using the paup* package (Swofford, 2003) with the best-fit GTR+Γ4 model of nucleotide substitution, determined by using modeltest (Posada & Crandall, 1998), and 1000 bootstrap neighbour-joining replications under the same ML model (the sequence alignment is provided as a nexus file in JGV Online). This analysis revealed that five patients were infected with genotype I viruses, whilst six patients were infected with viruses of genotype II (tree not shown; available from authors on request). However, it was not possible to obtain an unambiguous consensus nucleotide sequence, using cDNA as a template, for the E gene of a single virus isolate – D1.New Caledonia.416/03 – taken from a patient who developed dengue in New Caledonia in February 2003 (chromatogram available in JGV Online as Supplementary Fig. S1; all other isolates gave unambiguous sequences). Therefore, cDNA corresponding to the E genes of viruses from this patient was derived by RT-PCR using primers D1-764F and D1-2467R or D1-938F and D1-2386R (used to prepare clones D1.New Caledonia.416.3/03, 10/03, 14/03 and 91/03) and purified from 1.5 % (w/v) agarose–Tris/acetate EDTA gels by using a High Pure PCR purification kit (Roche) according to the manufacturer's instructions. 5′-ATP extensions were added to the cDNA (Craig *et al.*, 2003) and it was ligated into pGEM-T Easy plasmids (Promega) and used to transform *Escherichia coli* JM109 or DH5*α* bacteria (Sambrook & Russell, 2001). Plasmids were purified from individual bacterial colonies, which grew on Luria–Bertani agar supplemented with 100 μg ampicillin ml^−1^, 0.5 mM IPTG and 80 μg 5-bromo-4-chloro-indoyl-*β*-d-galactosidase ml^−1^ (Sambrook & Russell, 2001), using a commercial plasmid purification kit (Qiagen).

The nucleotide sequences of 24 clones derived from the virus population of D1.New Caledonia.416/03 (including seven obtained directly from serum and denoted ‘416S’ in all figures) varied at 266 sites. Clones 19 and 32 had single-nucleotide deletions at E250 and E308, respectively, resulting in frame-shifts and the production of downstream stop codons. The deduced amino acid sequences of the clones varied at 110 sites (after replacement of the nucleotide deletions in the sequences of clones 19 and 32). Clone 32 also had a stop codon (UAG) at amino acid position E248, as did clone 7 at E391.

Notably, some sequences of the clones from patient D1.New Caledonia.416/03 segregated with both genotype I and genotype II DENV-1, which were circulating in New Caledonia during the same outbreak, whereas others occupied ‘intermediate’ phylogenetic positions between the two genotypes, suggestive of recombination (Figs 1–3). To determine whether recombination had occurred among the viruses sampled from patient D1.New Caledonia.416/03, we employed the rdp2 program (Martin *et al.*, 2005), which employs six different algorithms to identify recombinants and their break points: rdp, SiScan, bootscan, chimeric, MaxChi and geneconv. Default settings were used in all cases. Strikingly, all six methods identified frequent recombination events; for example, geneconv, which is considered one of the most robust methods for detecting recombination (Posada, 2002), identified 1609 possible recombination events. Among all six methods in rdp2, there was particularly strong evidence for recombination break points at nucleotide positions 570, 620 and 1100.

To test further for recombination, ML phylogenetic trees were inferred for the regions of nucleotide sequence on either side of the break points detected by rdp2 using the methods described above (Figs 1–3). This analysis provided compelling evidence for recombination among the genotype I and II viruses that co-infected patient D1.New Caledonia.416/03, involving clones 1 (recombination event 1), 19 (recombination event 2) and 22 (recombination event 3). For each of these three recombinants, there was strong evidence for the phylogenetic incongruence indicative of recombination; the viruses occupied different phylogenetic (i.e. genotypic) positions on either side of specific break points that were supported by high numbers of bootstrap replications. Although clones 1, 19 and 22 represent clear instances of inter-genotype recombination, the extremely high frequency of recombination identified by rdp2, as well as the changing phylogenetic positions of other sequences (for example, clones 18 and 32 in Fig. 3), suggests that this process has occurred very frequently in this patient. Crucially, none of the break points were in regions containing sequences the same as, or complementary to, the sequences of oligonucleotide primers used for sequencing or for PCR, indicating that they are bona fide recombinants rather than laboratory artefacts.

As less than 50 μl (badly degraded) serum was available from patient D1.New Caledonia.416/03 after virus isolation had been performed, only seven clones of the E protein gene could be obtained from this source. The sequences of these clones served to confirm the presence of genotype 1 DENV-1 in this patient, but as none were recombinant, we were unable to distinguish whether the recombinant genomes that we detected in this virus isolate arose in the patient or during the isolation of the virus in cultures of *A. albopictus* mosquito cells. Indeed, it is highly likely that C6-36 cells amplify some subpopulations of virus selectively. Nonetheless, our results provide unambiguous evidence of intra-serotypic recombination in populations of DENV-1, if not in a patient, then at least in cells derived from a species of mosquito involved in dengue transmission. However, we are unable to determine whether these recombinants are viable, as it not possible to plaque-purify DENV to homogeneity. Even if the recombinant E protein is functional, nucleotide insertions and deletions elsewhere in the genome could render the virus non-functional.

This is only the second observation of both parental and recombinant DENV in a population within a single host (Craig *et al.*, 2003). Whilst there are several reports of infections of humans with multiple DENV serotypes, most techniques employed to identify virus in patient tissues would not detect infections with multiple genotypes of the same DENV serotype, as is clearly necessary to give rise to the recombinant viral populations described here. We therefore propose that recombination events in DENV may occur more frequently than has been reported, but that the practice of determining consensus nucleotide sequences from cDNA greatly limits the chances of their detection; only if the recombinant represents the dominant viral population will it be sampled by consensus sequencing. Further, nucleotide deletions, which also may be the footprints of recombination events, have been observed in a number of DENV populations (Aaskov *et al.*, 2006; Craig *et al.*, 2003; Lin *et al.*, 2004; Wang *et al.*, 2002). Hence, the examination of a much larger number of recombinant viruses and their parents may enable ‘hot spots’ of recombination to be identified and permit a systematic analysis of the factors mediating recombination in these viruses (Nagy & Simon, 1997).

By continuing to monitor the epidemiology of the ongoing outbreak of DENV-1 virus infection in the Pacific, it may be possible to determine whether the recombination events described here have conferred any fitness benefit on these viruses, although it is likely that most recombinants are deleterious and so removed by purifying selection. This notwithstanding, it is remarkable that both parental and different recombinant viral genomes were observed in an isolate from a single patient, particularly as the recombination appeared to have involved two genotypes of DENV-1 imported into New Caledonia (A-Nuegoonpipat *et al.*, 2004). This suggests that recombination in DENV may be more common than thought previously, but that the process will remain largely undetected until more studies of clonal diversity are undertaken.

## Supplementary Material

[Supplementary material]

## Figures and Tables

**Fig. 1. f1:**
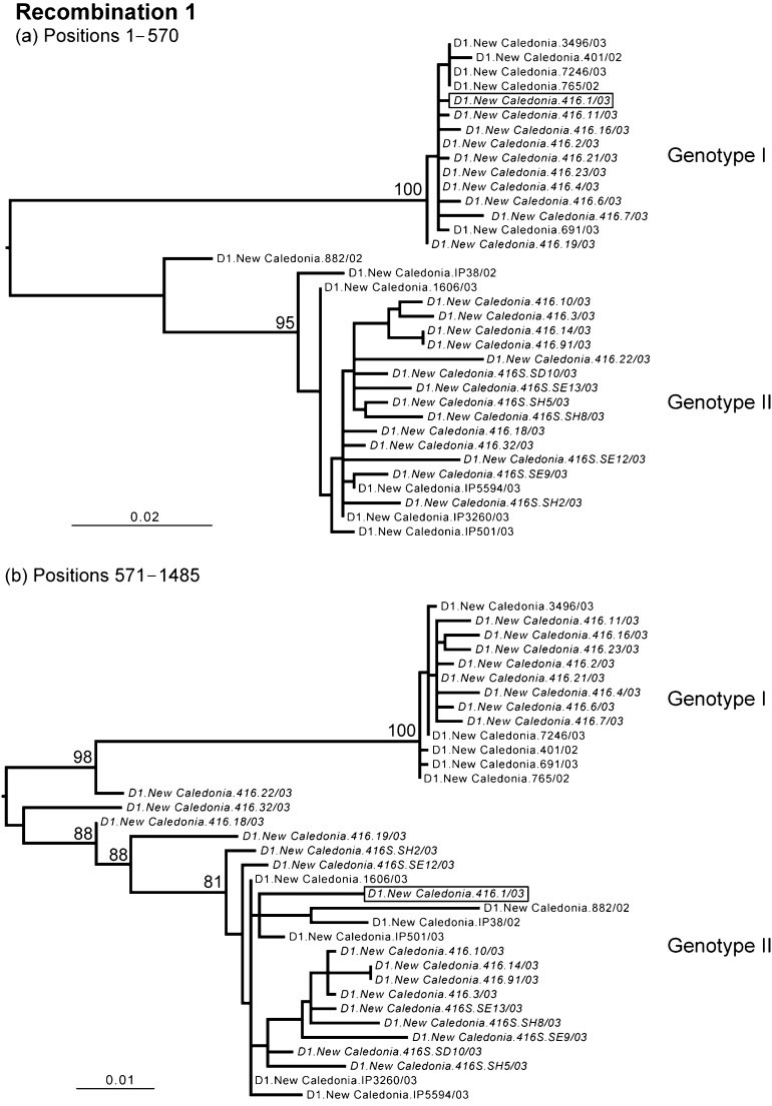
The changing phylogenetic position of clone D1.New Caledonia.416.1/03 (boxed), indicative of inter-genotypic recombination in DENV-1. The maximum-likelihood phylogenetic tree was inferred by using all (*n*=24) clones from patient D1.New Caledonia.416/03 (in italics), as well as the consensus sequences from 11 other patients sampled from New Caledonia, inferred for the entire E gene (1485 bp). Clones derived from serum are denoted ‘416S’. Bootstrap values are shown for key nodes only and all branches are scaled according to the number of nucleotide substitutions per site.

**Fig. 2.**
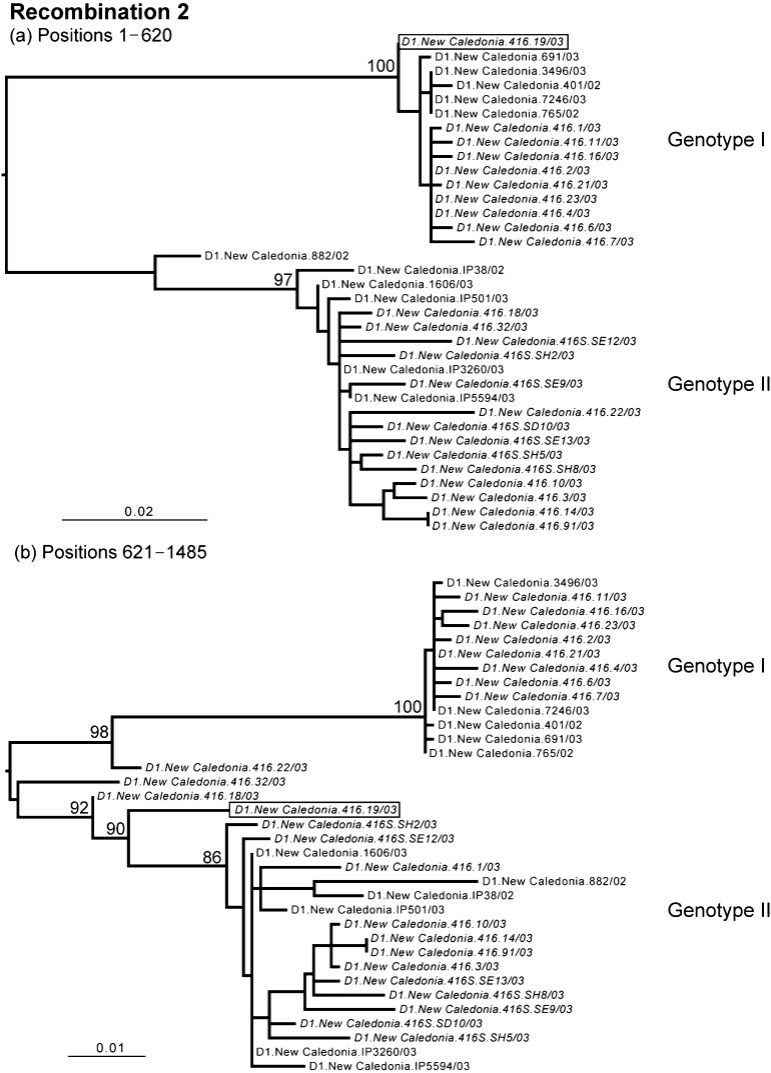
The changing phylogenetic position of clone D1.New Caledonia.416.19/03 (boxed), indicative of inter-genotypic recombination in DENV-1 (sequence labels as defined in the legend to Fig. 1). Bootstrap values are shown for key nodes only and all branches are scaled according to the number of nucleotide substitutions per site.

**Fig. 3. f3:**
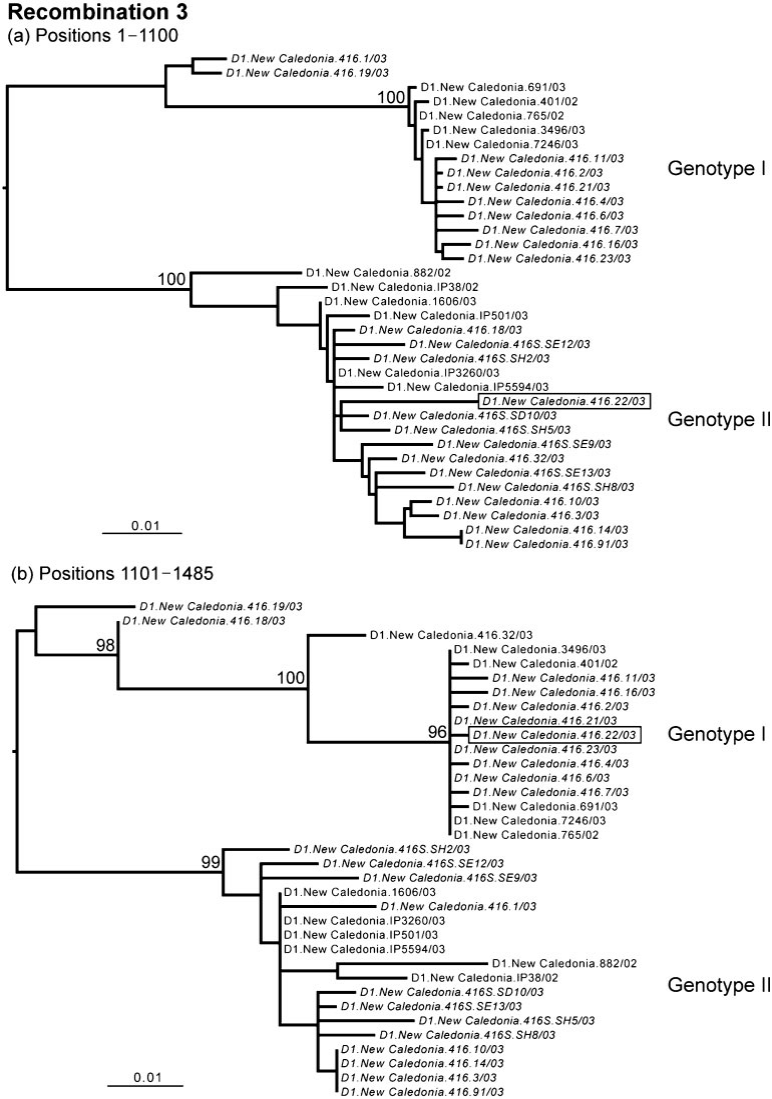
The changing phylogenetic position of clone D1.New Caledonia.416.22/03 (boxed), indicative of inter-genotypic recombination in DENV-1 (sequence labels as defined in the legend to Fig. 1). Bootstrap values are shown for key nodes only and all branches are scaled according to the number of nucleotide substitutions per site.
