# Supplementary material for: Multiple recombinant dengue type 1 viruses in an isolate from a dengue patient
Source: J Gen Virol. 2007 Dec;88(Pt 12):3334–40. doi: 10.1099/vir.0.83122-0 (PMC2884982; doi:10.1099/vir.0.83122-0)
Supplement: [Supplementary material] [file supp_88_12_3334__index.html]

 Multiple recombinant dengue type 1 viruses in an isolate from a dengue patient -- Aaskov et al. 88 (12): 3334 Data Supplement - Supplementary material -- Journal of General Virology

### Multiple recombinant dengue type 1 viruses in an isolate from a dengue patient, by J. Aaskov, K. Buzacott, E. Field, K. Lowry, A. Berlioz-Arthaud and E. C. Holmes

*Journal of General Virology* vol. **88**, part 12, pp. 3334 – 3340

**Supplementary Table S1.** Oligonucleotide primers used for PCR or sequencing of the DENV-1 E genes

**Supplementary Fig. S1.** Chromatogram of the sequence of complementary-strand cDNA obtained following RT-PCR

[Single PDF file] (401 KB)

**Alignment file** (NEXUS format) [alignment.nex]

---

|  |  |  |
| --- | --- | --- |
| INT J SYST EVOL MICROBIOL | MICROBIOLOGY | J GEN VIROL |
| J MED MICROBIOL | ALL SGM JOURNALS | |
