# Supplementary material for: Multiple recombinant dengue type 1 viruses in an isolate from a dengue patient
Source: J Gen Virol. 2007 Dec;88(Pt 12):3334–40. doi: 10.1099/vir.0.83122-0 (PMC2884982; doi:10.1099/vir.0.83122-0)
Supplement: [Supplementary material] [file supp_88_12_3334__1.pdf]

Supplementary Table S1. Oligonucleotide primers used for PCR or sequencing of the DENV-1 E genes

Primer*	Sequence (5′–3′)	Location†
T7	TAATACGACTCACTATAGGG	Plasmid
D1-764F	CAAATACAAAAAGTGGAGACCTGGGC	777–802
D1-938F	CGATGCGTGGGAATAGGCAGCATG	938–962
D1-789F	CTCTGAGACACCCAGGATTCAC	802–823
D1-843F	GCACATGCCATAGGAACATCC	843–863
D1-1391F	GCAACCATAACACCTCAAGC	1404–1423
D1-2386R	CCATGACTCCTAAGTATAGTGTTACTAG	2386–2413
D1-2467R	GACTTCATTGGTGACAAAAATGCCGC	2452–2477
SP6	GATTTAGGTGACACTATAG	Plasmid

*F, Sense primer; R, antisense primer.

†The positions of nucleotides in the genome of DENV-1 are numbered according to Fu *et al.* (1992).

Reference

Fu, J., Tan, B.-H., Yap, E.-H., Chan, Y.-C. & Tan, Y. H. (1992). Full length cDNA sequence of dengue type 1 virus (Singapore strain S275/90). *Virology* **188**, 953–958. Medline

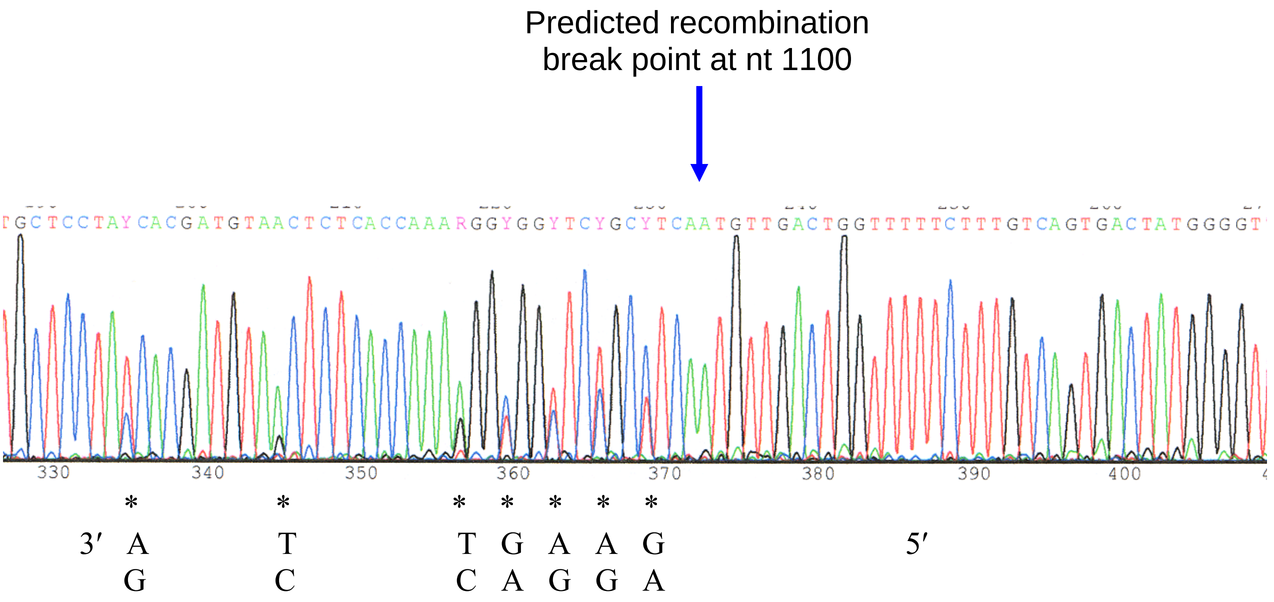

Supplementary Fig. S1. Chromatogram of the sequence of complementary-strand cDNA obtained following RT-PCR, showing ambiguous sequence adjacent to the third predicted recombination breakpoint. Clones 1,18, 19 and 32 had the nucleotides in the upper row at the sites indicated and clones 2,4, 6, 7, 11, 21 and 23 had the nucleotides in the lower row at these sites.

Aaskov, J., Buzacott, K., Field, E., Lowry, K., Berlioz-Arthaud, A. & Holmes, E. C. (2007). Multiple recombinant dengue type 1 viruses in an isolate from a dengue patient. *J Gen Virol* **88**, 3334–3340.
